# Ursodeoxycholic Acid but Not Tauroursodeoxycholic Acid Inhibits Proliferation and Differentiation of Human Subcutaneous Adipocytes

**DOI:** 10.1371/journal.pone.0082086

**Published:** 2013-12-03

**Authors:** Lucia Mališová, Zuzana Kováčová, Michal Koc, Jana Kračmerová, Vladimír Štich, Lenka Rossmeislová

**Affiliations:** 1 Department of Sport Medicine, Third Faculty of Medicine, Charles University in Prague, Prague, Czech Republic; 2 Franco-Czech Laboratory for Clinical Research on Obesity, Third Faculty of Medicine, Prague, Czech Republic; 3 INSERM, Toulouse, France; Beckman Research Institute of City of Hope, United States of America

## Abstract

Stress of endoplasmic reticulum (ERS) is one of the molecular triggers of adipocyte dysfunction and chronic low inflammation accompanying obesity. ERS can be alleviated by chemical chaperones from the family of bile acids (BAs). Thus, two BAs currently used to treat cholestasis, ursodeoxycholic and tauroursodeoxycholic acid (UDCA and TUDCA), could potentially lessen adverse metabolic effects of obesity. Nevertheless, BAs effects on human adipose cells are mostly unknown. They could regulate gene expression through pathways different from their chaperone function, namely through activation of farnesoid X receptor (FXR) and TGR5, G-coupled receptor. Therefore, this study aimed to analyze effects of UDCA and TUDCA on human preadipocytes and differentiated adipocytes derived from paired samples of two distinct subcutaneous adipose tissue depots, abdominal and gluteal. While TUDCA did not alter proliferation of cells from either depot, UDCA exerted strong anti-proliferative effect. In differentiated adipocytes, acute exposition to neither TUDCA nor UDCA was able to reduce effect of ERS stressor tunicamycin. However, exposure of cells to UDCA during whole differentiation process decreased expression of ERS markers. At the same time however, UDCA profoundly inhibited adipogenic conversion of cells. UDCA abolished expression of PPARγ and lipogenic enzymes already in the early phases of adipogenesis. This anti-adipogenic effect of UDCA was not dependent on FXR or TGR5 activation, but could be related to ability of UDCA to sustain the activation of ERK1/2 previously linked with PPARγ inactivation. Finally, neither BAs did lower expression of chemokines inducible by TLR4 pathway, when UDCA enhanced their expression in gluteal adipocytes. Therefore while TUDCA has neutral effect on human preadipocytes and adipocytes, the therapeutic use of UDCA different from treating cholestatic diseases should be considered with caution because UDCA alters functions of human adipose cells.

## Introduction

Obesity develops when the storage of surplus energy requires excessive expansion of the adipose tissue (AT). Expansion of AT occurs through hyperplasia or hypertrophy that is in adult obesity prevailing. Hypertrophy of adipocytes is connected with their dysfunction manifested by lower insulin sensitivity, higher basal lipolysis and altered production of cytokines contributing to a development of chronic low-grade inflammation [1,2]. Even though the exact molecular insult leading to such adipocyte dysfunction is not clear, it appears that the nutrient overload creating excessive demands on the endoplasmic reticulum (ER) could be an important if not central contributor [3,4]. ER is an organelle with the direct control over the cytokine production and lipid storage and its overload initiates processes that should enhance ER capacity but also potentiate typical pro-inflammatory pathways [5]. Indeed, ER stress (ERS) is higher in obese insulin resistant subjects that at the same time show evidence of low grade inflammation [6,7]. On the other hand, the resolution of ERS by chemical chaperones has been shown to alleviate inflammation [5,8]. One class of the chemical chaperones is represented by bile acids (BAs), natural products of cholesterol catabolism [9]. BAs were shown to prevent ERS in AT of obese mice [10]. Apart from their chaperone capacity, BAs may influence metabolic state of AT also by regulating other pathways as evidenced by animal studies, i.e. BAs were shown to regulate adipocyte functions through the activation of nuclear farnesoid X receptor (FXR) and specific G protein-coupled membrane surface receptor TGR5 [11,12]. In 3T3-L1 cells, FXR cooperates with PPARγ and in addition to that it stimulates adipogenesis also through inhibition of Wnt pathway [11,13].In brown adipocytes, TGR5 pathway regulates energy expenditure through the induction of mitochondrial uncoupling protein (UCP1) expression [12]. However, these findings have not yet been confirmed in humans and effects of BAs on properties of human preadipocytes, resp. adipocytes remain mostly unknown. Indeed, this study aimed to evaluate and compare the effects of two common species of BAs, ursodeoxycholic (UDCA) and tauroursodeoxycholic acid (TUDCA), on proliferation and adipogenic conversion of human preadipocytes as well as on their inflammatory status. Since adipocytes characteristics differ in respect to the fat depot, the effects of BAs were evaluated in cells derived from abdominal (sAAT) and gluteal (sGAT) subcutaneous AT.

## Materials and Methods

### Subjects

10 premenopausal obese women (body mass index [BMI] 32.8 ± 3.2 kg/m^2^) without medication and diseases except for obesity participated in this study. The written informed consent was obtained from each patient before the study. The study was performed according to the Declaration of Helsinki protocols and was approved by Ethical Committee of the Third Faculty of Medicine, Charles University in Prague.

### Clinical investigation and laboratory measurements

Complete clinical investigation including anthropometric measurements, blood sampling and AT biopsies was performed in the morning in the fasting state. The whole body composition was evaluated by multi-frequency bioimpedance (Bodystat, Quad scan 4000, Isle of Man, UK). The blood was collected and centrifuged at 1300 RPM, 4°C, separated plasma was stored at -80°C until analysis. The paired samples of subcutaneous AT were obtained from the subcutaneous abdominal (10 cm lateral to the umbilicus) and gluteal (right upper quadrant) region using needle biopsy under local anesthesia (1% Xylocaine). Plasma glucose was determined using the glucose-oxidase technique (Beckman Instruments, Fullerton, CA). Plasma insulin was measured using an Immunotech Insulin Irma kit (Immunotech, Prague, Czech Republic). Homeostasis model assessment of the insulin resistance index (HOMA-IR) was calculated as follows: ((fasting insulin in mU/l) x (fasting glucose in mmol/l) / 22.5). Plasma levels of other relevant substances were determined using standard clinical biochemistry methods. Anthropometrical and biochemical characteristics of subjects are shown in Table 1.

**Table 1:** Clinical characteristics of subjects.

**Age (years)**	43±0.7
**BMI (kg/m^2^)**	32.8±0.3
**Weight (kg)**	91.3 ± 1.1
**Waist circumference (cm)**	100.9±1.0
**Hip circumference (cm)**	120.1±0.9
**Fat mass (%)**	41.0±0.6
**FFM (%)**	59.1±0.6
**Glucose (mmol/l)**	5.2 ±0.1
**Insulin (mIU/l)**	8.9±0.5
**NEFA (mmol/l)**	0.5±0.0
**Triglycerides (mmol/l)**	1.2±0.1
**HDL cholesterol (mmol/l)**	1.4±0.0
**Total cholesterol (mmol/l)**	4.7±0.1
**HOMA–IR**	2.1±0.1

Values are means ± SEM, n = 10.

BMI, body mass index; FFM, fat-free mass; HOMA-IR, homeostasis model assessment of the insulin resistance index; NEFA, nonesterified fatty acids, HDL, high-density lipoprotein

### Isolation, cultivation and differentiation of preadipocytes

Samples of AT were washed with PBS supplemented with gentamycin and then digested with collagenase I (300 U/ml, Biochrom, Berlin, Germany) for 40-60 minutes in shaking water bath at 37°C. The digested AT was centrifuged twice (1300 RPM, 5 min), adipocytes were discarded and pellet containing stroma-vascular fraction (SVF) was incubated in erythrocyte lysis buffer for 10 min at room temperature. Cells were collected by centrifugation and, without any filtration step, they were resuspended in PM4 medium (base medium-DMEM/F12, L-glutamine, Panthotenate, biotin, gentamycin, Pen/strep, supplemented with 2.5% MSC qualified FBS, Invitrogen, 1 ng/ml FGFβ, 10 ng/ml EGF, 132 nM insulin). The proliferation medium (PM4) was change every 2 days until the cells reached 70% confluence, then they were subcultivated. Two additional subcultivations were performed and then cells (passage 4) were plated for proliferation assay at density specified bellow or 10 000 cells/cm^2^ for experiments on differentiated adipocytes.

To induce adipogenic differentiation, two days postconfluent cells were washed with PBS with Ca^2+^/Mg^2+^ and fed with DIFM+ medium (base medium supplemented with 2.5% MSC qualified FBS, Invitrogen, 66 nM insulin, 1 µM dexamethasone, 1 nM T3, 0.1 µg/ml transferin, 0.25 mM IBMX, 1 µM Rosiglitazone). The medium was changed after 3 days. At day 6^th^, Rosiglitazone and IBMX were omitted and dexamethasone replaced with 0.1 µM cortisol. The differentiation continued until day 12 with one change of media. 

For experiments with BAs, proliferation and differentiation medium were supplemented with 200 µM UDCA (Sigma Aldrich, St Louis, MO, USA), 500 µM TUDCA (Calbiochem, San Diego, CA, USA) or PBS as control. The effective concentrations of BAs were based on previously published observations [14,15]. To create ERS, cells were treated with 1 mg/ml tunicamycin (LKT Laboratories, St. Paul, MN, USA). To activate FXR, cells were treated with 10 µM GW4064 (Sigma Aldrich). To activate NFκB pathway, cells were treated with 10 ng/ml TNFα (Immunotools, Friesoythe, Germany). To prevent phosphorylation of Erk1/2, cells were pretreated with 50 µM PD98059 (Enzo, Farmingdale, NY, USA).

### Proliferation assay

MTS assay-2000 cells/cm^2^ were plated onto 96 well plate, in triplicates for each condition and cultivated in PM4 medium supplemented with either UDCA, TUDCA or PBS. Medium was changed on day 2 and 4. BAs were present in medium during the whole proliferation assay. Numbers of adherent cells were estimated using MTS assay (CellTiter96 aqueous MTS reagent powder, Promega, Madison, WI, USA; Phenazine methosulfate, Sigma) by assessing the absorbance of formazan measured at 490 nm.

Cell cycle - Cells were cultured and treated as described for proliferation assays. At day 5, they were trypsinized and fixed in 70% ethanol at 4°C overnight. Then cells were washed with PBS two times, stained with 50 µg/ml Propidium Iodide and treated with 0.1 mg/ml RNAse I diluted in PBS for 30 minutes at 37°C. Cell cycle analysis was performed on FACSCalibur and analyzed with FlowJo 8.2 (BD Biosciences, Franklin Lakes, NJ, USA).

### Gene expression analysis

For RNA analysis, cells were lysed in RLT buffer and total RNA was isolated using RNeasy Mini Kit (Qiagen, Hilden, Germany). RNA concentration was measured by Nanodrop 1000 (Thermo Fisher Scientific, Wilmington, USA). Genomic DNA was removed by DNAse I treatment (Invitrogen, Carlsbad CA, USA). cDNA was obtained by reverse transcription (High Capacity cDNA Reverse Transcription Kit, Applied Biosystem, Carlsbad, CA, USA) of 600 ng of total RNA. cDNA equivalent of 5 or 25 ng of RNA was used for Real Time PCR reactions using Gene Expression Master Mix or Universal Master Mix II and Gene expression assay of PPARγ, SCD1, FASN, b2ADR, HSPA5, ATF4, FXR, TGR5, TLR4, GROα, MCP1, IL8, UCP1 (Applied Biosystem). aP2 was detected by specific primers by Sybr Green technology (Power Sybr Green Master Mix). All samples were run in duplicates on 7500 Fast ABI PRISM instrument (Applied Biosystem). Gene expression of target genes was normalized to expression of GUSB (glucuronidase, beta) or to same input of cDNA (in case of time course of differentiation when all tested control genes exhibited substantial shifts in Ct value) and fold change of expression was calculated using ΔΔ Ct method. 

### Oil Red O (ORO) staining

12 days differentiated cells were fixed by direct addition of buffered formaline into media (1v:1v), after 10 minutes medium was discarded and replaced by fresh undiluted formaline for another 20 minutes. Cells were washed several times in PBS and once with 60% isopropanol then stained with 60% ORO for 20 minutes. After extensive washing with water, ORO was eluted with 100% isopropanol and absorbance of eluates was measured at 500 nm. Standard curve from working stock of ORO was performed to normalize data and decrease inter-experimental variation.

### Western blotting

Cells were washed two times with PBS and lysed on ice for 30 minutes in RIPA lysis buffer supplemented with protease and phosphatase inhibitors (Complete, PhoStop, Roche Diagnostics, Mannheim, Germany). Lysates were then centrifuged for 15 minutes at 15,000x g, 4°C. Protein concentrations were determined using the bicinchoninic assay, Pierce (Rockford, IL, USA). Samples were loaded to a 10% acrylamide minigel and electrotransferred onto the nitrocellulose membrane. Membranes were blocked with 5% BSA. Antibodies against actin, IκBα, NFκB, Erk1/2 and their phosphorylated forms were from Cell Signaling (Danvers, MA, USA). Antigen-antibody complexes were detected using secondary antibodies coupled with horseradish peroxidase and the ECL detection system (Pierce).

### Statistical analysis

The data from RT-qPCR were analyzed with GraphPad Prism 5.0. (La Jolla, CA, USA). Wilcoxon paired t-test was used for comparison of gene expression of paired samples between sAAT vs. sGAT, the effect of various treatments separately in each depot was estimated by Main-Whitney test. The levels of significance was set at p<0.05. 

## Results

### Effect of BAs on proliferation of preadipocytes

**Figure 1:**
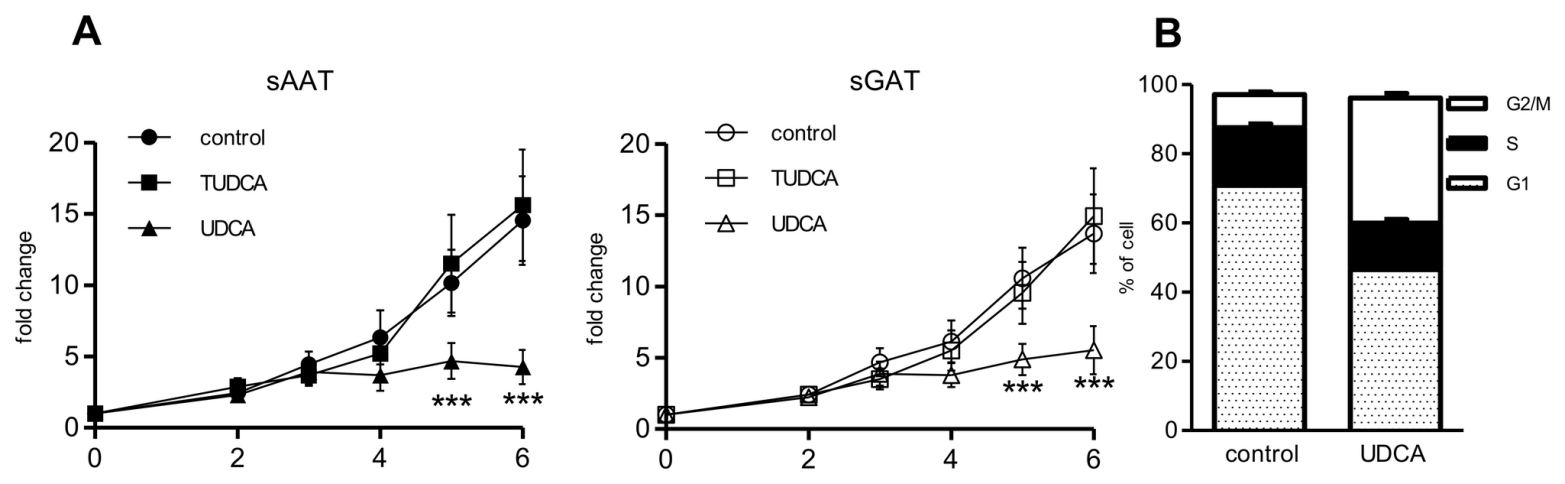
Effect of BAs on proliferation of preadipocytes. (A) Preadipocytes were seeded at density 2000 cells/cm^2^ and cultivated for 6 days under control conditions or in the presence of 200 µM UDCA or 500 µM TUDCA. MTS assay was performed at indicated days. Fold changes of measured absorbance over the control was calculated for each donor (n=10). Data are means ±SE, *** p<0.001. (B) Cell cycle analysis. sAAT preadipocytes were seeded at density 2000 cells/cm^2^ and cultivated for 5 days under control conditions or in the presence of 200 µM UDCA. Then cells were harvested, stained with propidium iodide and analyzed by flow cytometry (n=5). The percentage of cells in G1, S and G2/M cell cycle phases is shown.

To evaluate effect of BAs on preadipocytes and adipocyte properties we have established the cultures of preadipocytes from paired samples of sAAT and sGAT of 10 obese women. Under standard growth conditions, proliferation of preadipocytes from both depots was similar (Figure 1A). The concentration of formazan, read out of MTS proliferation assay, increased after 6 days of proliferation 14.53±3.11 times for sAAT and 13.7±2.76 times for sGAT preadipocytes. TUDCA did not affect proliferation of preadipocytes from either depot. UDCA had however strong anti-proliferative effects, as it almost completely blunted the division of cells from both depots. To investigate how UDCA inhibits growth, we analyzed the cell cycle of cells from sAAT after 5 days of growth under control condition or in medium supplemented with UDCA. Based on FACS analysis, percentage of apoptotic cells in control and UDCA-treated cells was negligible. However, the distribution of cells in cell cycle was substantially affected by UDCA, i.e. percentage of cells in G2/M phase increased from 9.55±0.75% to 36.16±1.3% , while percentage of cells in G1 phase decreased (control v. UDCA 70,91 ±0.44 v. 46.5±2.3) (Figure 1 B). 

### Effect of BAs on key ERS proteins

**Figure 2:**
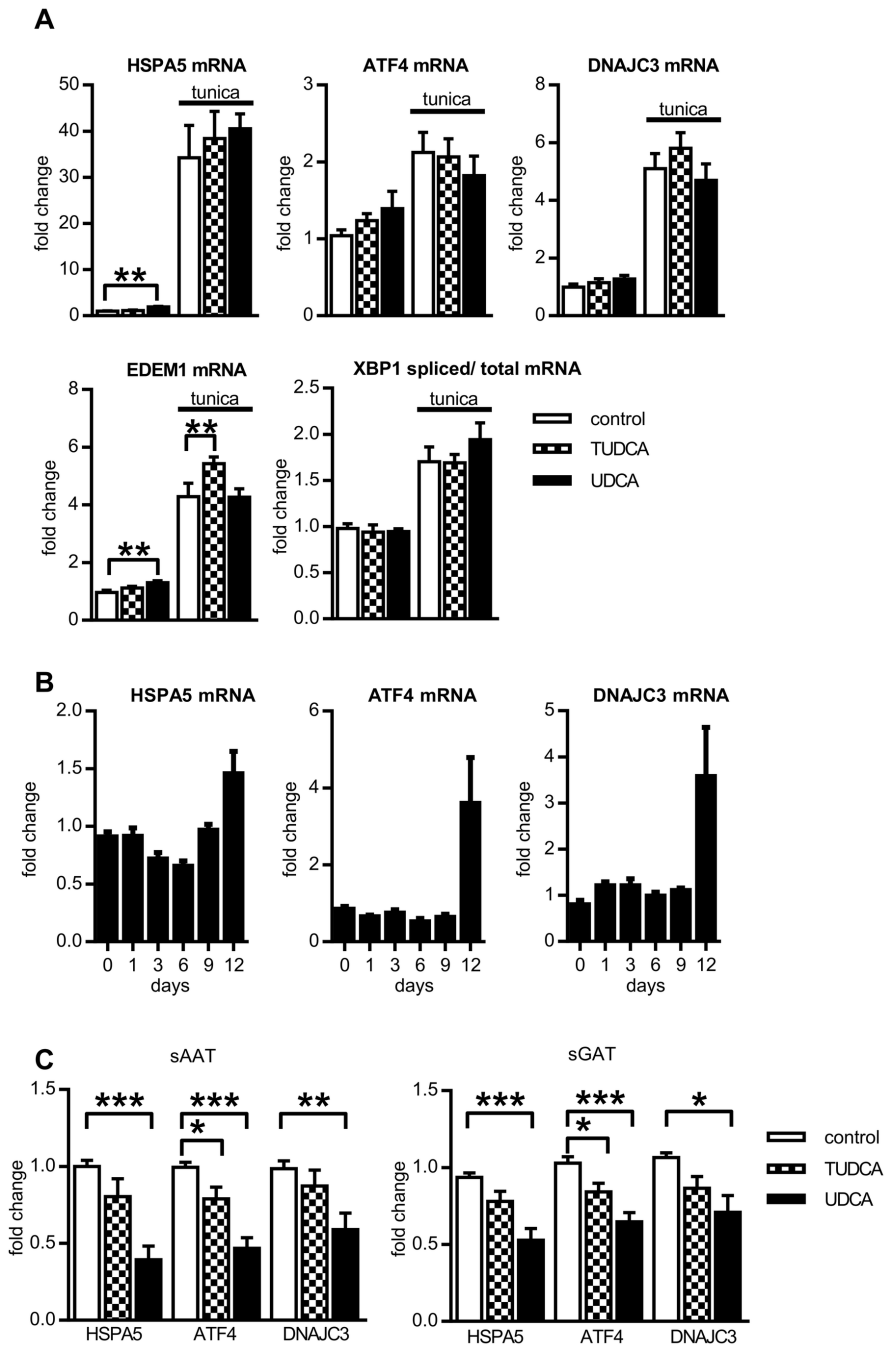
Effect of BAs on ERS markers. (A). Cells were differentiated for 12 days, and after 2 hr pretreatment with BAs they were exposed to 1µg/ml tunicamycin for 24 hrs. Then gene expression of ERS markers was analyzed and expressed as fold change in mRNA expression normalized to GUSB expression (n=3). (B) Cells were differentiated for indicated days and then gene expression of ERS markers was analyzed. Fold change in mRNA expression in adipocytes was normalized to input of cDNA (n=3) (C) Cells were differentiated for 12 days in the absence or presence of 200 µM UDCA or 500 µM TUDCA and then gene expression of ERS markers was analyzed. Fold change in mRNA expression in adipocytes was normalized to GUSB expression (n=10). Data are means ±SE, *p<0.05, *** p<0.001.

To confirm previously described potential of BAs to prevent development of ERS in adipocytes, in vitro differentiated human adipocytes pretreated with BAs for 2 hours were exposed to ERS inducer tunicamycin (1µg/ml) for additional 24 hours (BAs were still present in medium) and then, the mRNA expression of five ERS markers, HSPA5, ATF4, DNAJC3, XBP1 (spliced versus total) and EDEM1, representing targets of all three arms of unfolded protein response (UPR), was evaluated. Unexpectedly, pretreatment of adipocytes with BAs did not prevent upregulation of ERS markers mRNA induced by tunicamycin (Figure 2A). This prompted us to determine whether BAs exert effects on expression of ERS markers when present chronically during whole time course of adipogenesis. The process of adipogenic differentiation in the absence of BAs was accompanied with the modest increase of HSPA5 expression and upregulation of ATF4, DNAJC3 mRNA at the end of differentiation (Figure 2B). Differentiation in the presence of BAs decreased mRNA expression of ERS marker ATF4, but only UDCA lowered expression of HSPA5 and DNAJC3 (Figure 2C). At the same time, we noticed that UDCA affected the process of differentiation and this effect was studied in the subsequent series of experiments.

### Effect of BAs on adipogenic conversion of preadipocytes

**Figure 3:**
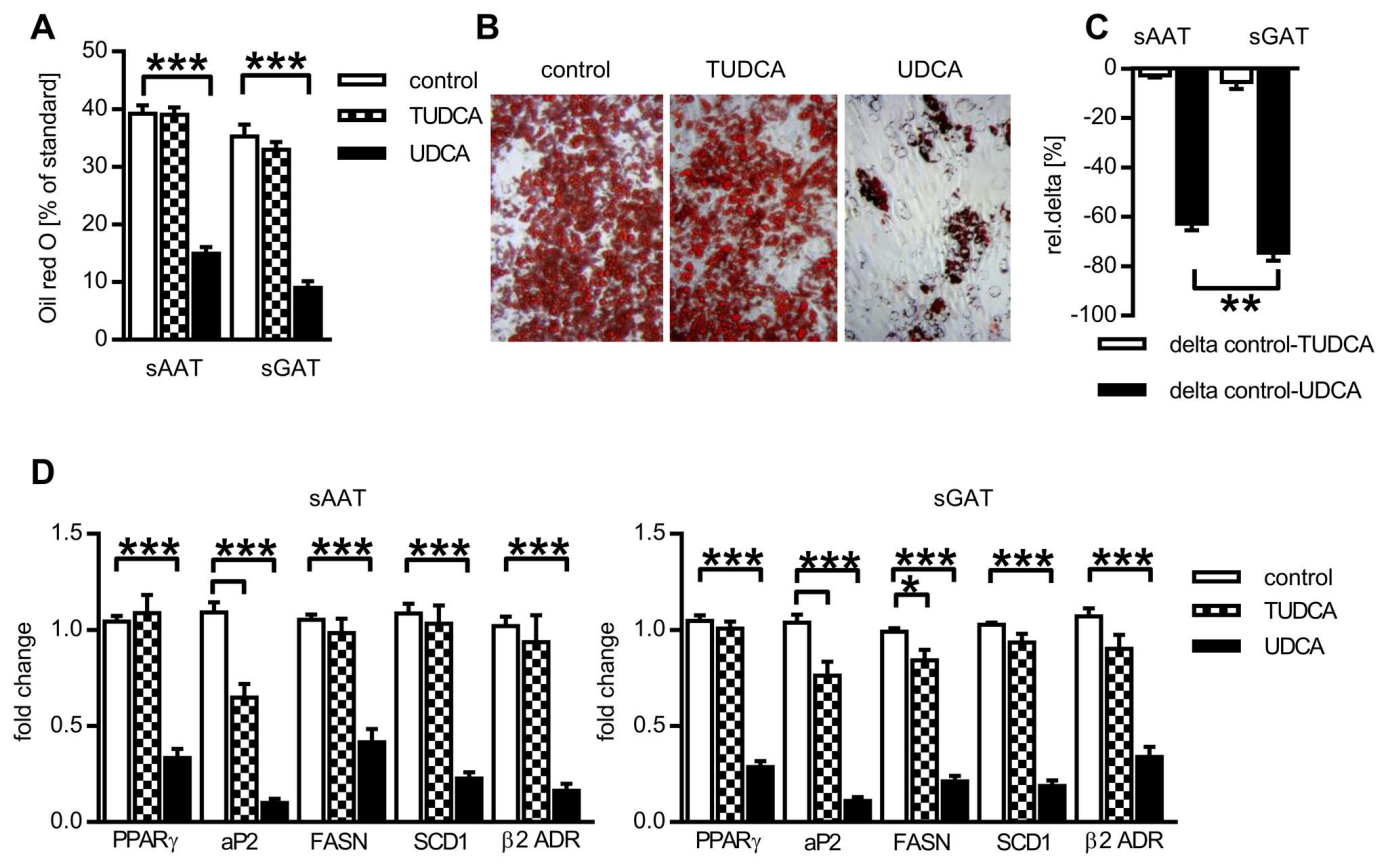
Effect of BAs on adipogenic differentiation of preadipocytes. Cells were differentiated for 12 days in the absence or presence of 200 µM UDCA or 500 µM TUDCA and then accumulation of lipids or gene expression was analyzed. (A) Effect of BAs on lipid accumulation. Quantification of neutral lipid accumulation expressed as a % of stock ORO (n=10). (B) Representative images of adipocytes from one donor stained with ORO. (C) Comparison of sensitivity of adipocytes from sAAT and sGAT depots to BAs treatment. Relative delta between control and BAs treatment was assessed for each donor (n=10). (D) Effect of BAs on gene expression in adipocytes. Fold change in mRNA expression in adipocytes was normalized to GUSB expression (n=10). Data are means ±SE, *p<0.05, ** p<0.01, *** p<0.001.

The ORO staining after 12 days of adipocytes cultivation with BAs showed that accumulation of neutral lipids in cells stimulated to adipogenesis was not altered by TUDCA but was substantially reduced by UDCA treatment when compared with control conditions (Figure 3 A, B). This anti-adipogenic potential of UDCA was more pronounced in preadipocytes derived from sGAT (Figure 3C). Inhibitory effect of UDCA on adipogenesis and lipogenesis was confirmed on mRNA levels of several genes typical for mature adipocytes- expression of two markers of differentiation process, PPARγ and aP2, two markers of lipogenesis, FASN and SCD1, and β2 adrenergic receptor was decreased after 12 days of UDCA treatment (Figure 3D). Notably, aP2 mRNA level was significantly decreased also in the presence of TUDCA in cells from both depots. 

### Effect of BAs on expression of BA receptors and activity of Erk1/2

**Figure 4:**
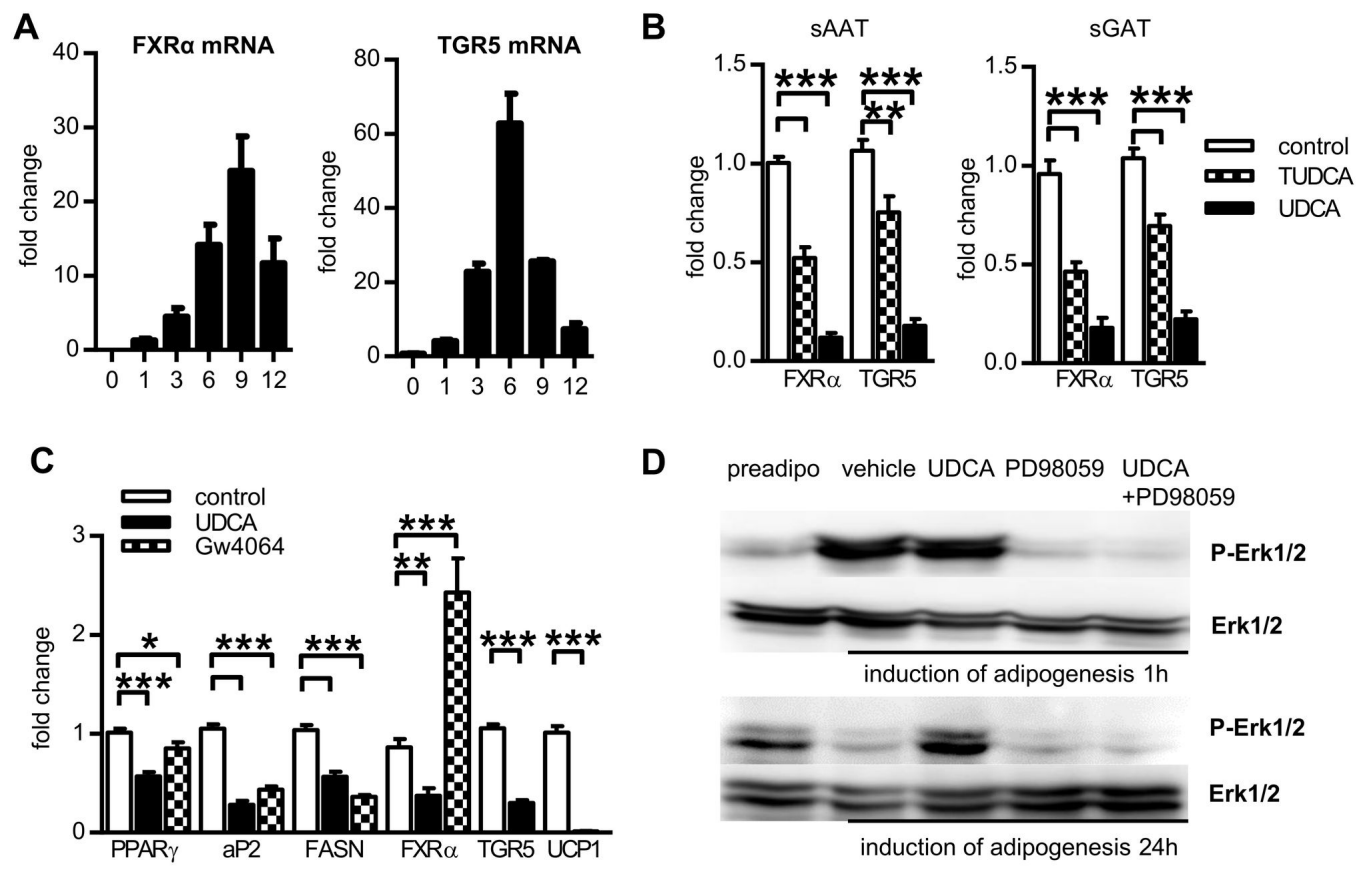
Expression of BA receptors and Erk1/2 in human adipocytes and their modulation by BAs. (A) Cells were differentiated for indicated days and then gene expression of FXRα and TGR5 was analyzed. Fold change in mRNA expression in adipocytes was normalized to input of cDNA (n=3) (B) Cells were differentiated for 12 days in the absence or presence of 200 µM UDCA or 500 µM TUDCA and then gene expression of FXRα and TGR5 was analyzed. Fold change in mRNA expression in adipocytes normalized to GUSB expression (n=10). Data are means ±SE, *** p<0.001. (C) Effect of UDCA and FXR agonist GW4046 on early phases of adipogenesis. Cells were induced to differentiate in the presence of 200 µM UDCA or 10 µM GW4046 for 3 days and then gene expression was analyzed. Fold change in mRNA expression in adipocytes was normalized to GUSB expression (n=3). Data are means ±SE, *p<0.05, ** p<0.01, *** p<0.001. (D) Western blotting analysis of Erk1/2 activation. Preadipocytes switched to adipogenic medium were treated with 200 µM UDCA and/or 50 µM PD98059 (PD98059 was added 30 minutes before the start of differentiation) for indicated times.

Based on the fact that UDCA can modulate activity of FXR and TGR5 - two known BAs receptors - we hypothesized that anti-adipogenic effect of UDCA might be mediated through these two factors. However, expression of neither FXRα nor TGR5 in human white adipocytes was reported previously. Therefore, we analyzed evolution of expression of FXRα and TGR5 during adipogenesis. FXRα mRNA was undetectable in preadipocytes but was induced during adipogenic conversion of cells (Figure 4A). TGR5 was expressed already in preadipocytes and its expression was strongly upregulated during the induction phase of differentiation, i.e. up to day 6, and then its mRNA levels decreased gradually (Figure 4A). When the adipocytes were differentiated in the presence of BAs, the expression of FXRαand TGR5 mRNA was suppressed in UDCA- and to lesser extent also in TUDCA-treated adipocytes from both depots (Figure 4B). To evaluate the involvement of FXR in UDCA-induced inhibition of adipogenesis, cells were induced to differentiate in the presence or absence of UDCA or specific FXR ligand GW4064 for 3 days when we expected major impact on the cascade of adipogenic transcription factors. UDCA treatment and the activation of FXR receptor with specific agonist GW4064 resulted in the similar suppression of expression of differentiation markers PPARγ, aP2 and FASN but had opposite effect on FXR expression itself (Figure 4C). 

Both, TUDCA and UDCA, can also activate TGR5 receptor. In brown AT, TGR5 activation leads to upregulation of UCP1 expression [12]. However, even though UCP1 was expressed in sAAT and sGAT adipocytes, its expression was not altered by TUDCA and strongly suppressed by UDCA (data not shown). Moreover, expression of both, TGR5 and UCP1, was significantly repressed as early as after 3 days after induction of adipogenesis (Figure 4C).

Another pathway putatively activated by UDCA and also capable to regulate early steps of adipogenesis involves Erk1/2 activation [16]. Sustained activation of Erk1/2 may lower PPARγ transcriptional potency [17]. Therefore, to evaluate immediate effects of UDCA on Erk1/2 phosphorylation levels, we exposed cells to adipogenic medium in the presence or absence of UDCA or PD98059 (Erk1/2 inhibitor) for 1 hour and 24 hours. The adipogenic medium itself and in combination with UDCA induced sharp increase in Erk1/2 phosphorylation levels that was prevented by pretreatment with PD98059. Phosphorylation degree of Erk1/2 returned to basal levels after 24 hrs in control cells while UDCA treated cells maintained high Erk1/2 phosphorylation (Figure 4D). 

### Effect of BAs on expression of cytokines

**Figure 5:**
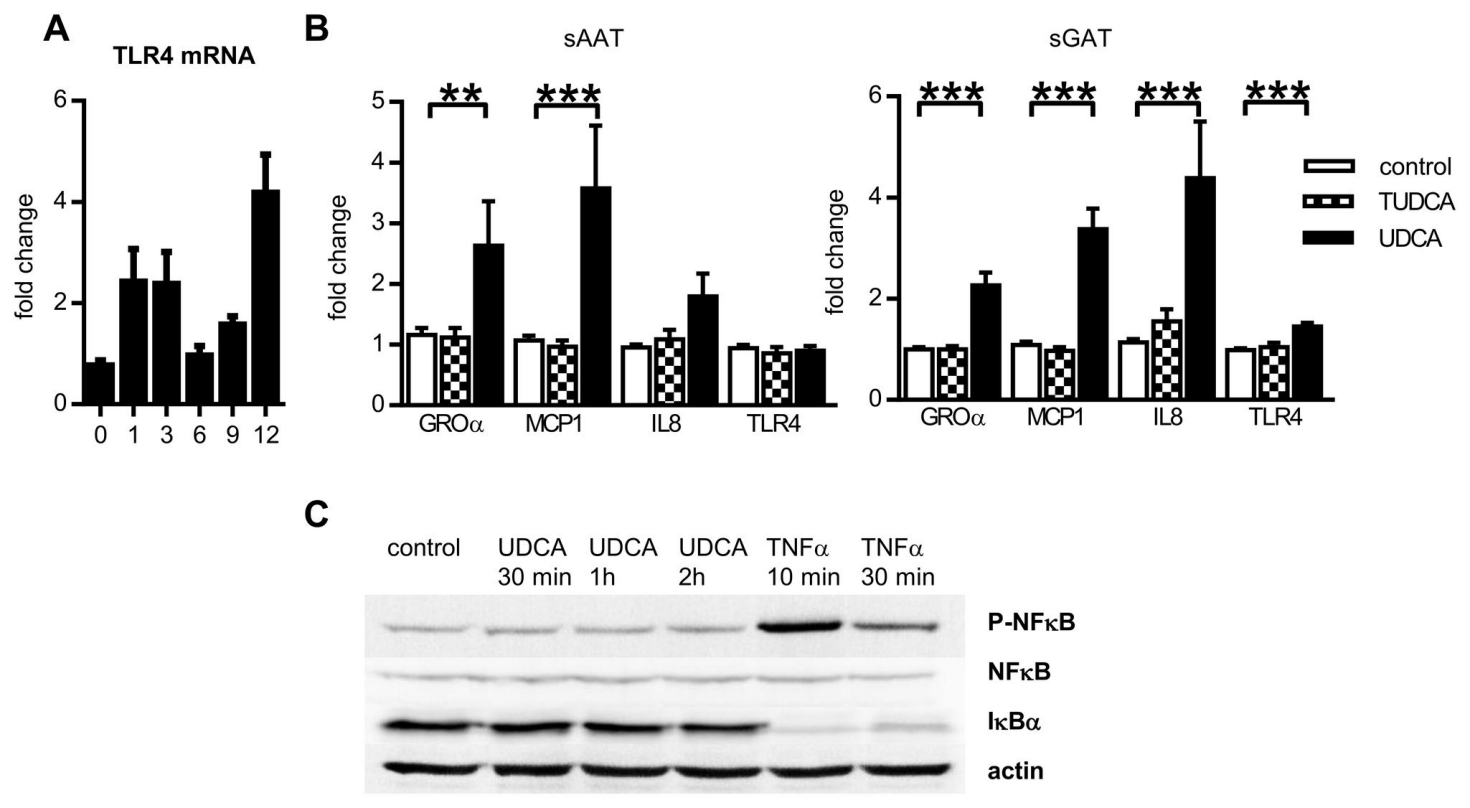
Effect of BAs on target genes of TLR4 pathway. (A) Cells were differentiated for indicated days and then gene expression of TLR4 was analyzed. Fold change in mRNA expression in adipocytes normalized to input of cDNA (n=3). (B) Cells were differentiated for 12 days in the absence or presence of 200 µM UDCA or 500 µM TUDCA. Fold changes in mRNA expression in adipocytes normalized to GUSB expression (n=10). Data are means ±SE, *p<0.05, ** p<0.01, *** p<0.001. (C) Western blotting analysis of NFκB activation. Preadipocytes were treated with 200 µM UDCA or 10 ng/ml TNFα (positive control) for indicated times.

Finally, we tested whether BAs can positively influence the inflammatory status of adipocytes since BAs were reported to have immunomodulatory properties [18]. Adipogenesis itself was connected with mild induction of TLR4 expression (Figure 5A). TLR4 pathway is coupled with activation of NFκB that is directly responsible for enhancement of cytokine expression stimulation. We hypothesized that adipocytes differentiated in the presence of BAs would exert lower basal expression of inflammatory cytokines regulated by TLR4 pathway, i.e. IL8 [19], GROα and MCP1 [20]. However, chronic supplementation of adipogenic medium with TUDCA did not decrease expression of GROα, IL8, MCP1 or TLR4. Moreover, differentiation of adipocytes in the presence of UDCA led to enhanced mRNA expression of GROα and MCP1 in adipocytes from both depots and of IL8 and TLR4 selectively in sGAT adipocytes (Figure 5B). Surprisingly, despite this pro-inflammatory potential of UDCA, exposition of preadipocytes to UDCA did not initiate phosphorylation of NFκB and degradation of IκBα (Figure 5C).

## Discussion

Chronic surplus of energy leading to development of obesity can, on cellular level, disrupt the function of ER and thus induce ERS. ERS is suggested as one of the first steps leading to deterioration of AT functions [5]. Thus, it was hypothesized that improvement of ER function in stressed adipocytes could restore their metabolic and endocrine profile to pre-obese conditions. Two naturally occurring BAs, UDCA and TUDCA, are considered as potent alleviators of ERS as they prevent aggregation of proteins and inhibit activation of UPR pathway [21-23]. Importantly, both UDCA and TUDCA are powerful detergents with low toxicity, and as such they have been already approved for clinical use for treatment of several cholestatic diseases [24]. However, BAs may modulate the metabolic and endocrine function of AT not only through their action on ER but also through direct activation of BA-specific receptors. Thus, this study evaluated effects of UDCA and TUDCA on several characteristics of human adipose cells derived from two subcutaneous depots, which differ in their metabolic function [25,26] and type of expansion [27].

Proliferation of preadipocytes is prerequisite for maintenance and also hyperplasic expansion of AT. In adulthood, hyperplasia is stimulated preferentially in sGAT, whereas adipocytes from sAAT depot become in response to excessive caloric intake rather hypertrophic [27]. Therefore we tested effects of BAs on proliferation of preadipocytes isolated from these two depots. Our data showed that although TUDCA had no apparent effect on proliferation of preadipocytes, UDCA exhibited unexpectedly strong anti-proliferative potential. UDCA was previously shown to inhibit proliferation of several types of carcinoma cells [28] as well as the growth of normal intestinal cells in vivo when administered to mice for 3 weeks [16]. Anti-proliferative effect of UDCA was associated with cell cycle arrest, apoptosis or cell senescence [29-31]. Our FACS analysis excluded apoptosis as possible mechanism of UDCA anti-proliferative effect in preadipocytes (Figure 1 C). Moreover, we did not observe an increased appearance of senescent cells with large nuclei and flattened morphology following the UDCA treatment. On the other hand the substantial enrichment of G2/M peak supports previous observations showing that UDCA can block cell cycle progression at the G2/M phase [32]. 

The sensitivity to anti-proliferative effects of UDCA as well as the proliferation under standard culture conditions was not different between preadipocytes from the two depots. Thus the increased proliferation of sGAT preadipocytes observed in vivo during overfeeding [27] is probably dependent more on the local tissue milieu than on the intrinsic properties of cells derived from this depot. 

The chaperone like property of BAs observed in several cellular and animal models [10,22,33] indicated BAs as a potential therapy of obesity-associated comorbidities. However, we could not confirm this beneficial effect of BAs in human adipocytes as neither of BAs was able to alleviate acute ERS induced by tunicamycin in these cells. Indeed, in support of our observation in human adipocytes, in vivo treatment of obese subjects with TUDCA did not alter the level of ER chaperone expression in AT [34]. It is assumed that adipocytes cannot readily uptake certain BAs because expression of transporters responsible for BAs tissue uptake is very low in most extrahepatic tissues [35,36]. In fact, BAs are effectively metabolized by the liver, intestine and other cells that are naturally exposed to BAs [9] but little is known about metabolism of BAs in adipose cells. In addition, in the study of Berger et al. [21] proving BAs protective effect in epithelial cells, supraphysiological amount of TUDCA were used (1-10 mM) compared to 200 and 500 µM UDCA and TUDCA resp. used in this study. Thus, it is possible that higher concentration or prolonged exposition of already differentiated adipocytes to BAs might be needed to achieve adequate effect on ERS levels. In fact, the prolonged exposition of cells to UDCA did lower expression of HSPA5, ATF4 and DNAJC3 (Figure 2C). This UDCA treatment, however, blocked adipogenic conversion of adipose precursors. 

The distinct effects of TUDCA and UDCA on both ER proteins and adipogenesis could be based also on the fact that UDCA and TUDCA differ in their hydrophility and affinity to receptors. TUDCA cannot pass the cellular membrane of cells lacking specific transporters [35]. Major effects of TUDCA in cells of non-hepatic origin could be therefore ascribed to its binding to TGR5 receptor [35]. UDCA, on the other hand, can readily diffuse through membranes and apart from TGR5 [37] can weakly activate also nuclear FXR [38]. Both, TGR5 and FXR, seem to play important role in AT physiology. FXR was implicated in the regulation of the AT insulin sensitivity and of adipocytes differentiation and function [11,13,39]. Although expression of FXR in human adipose cells was not detected [40], we confirmed that FXRα was expressed in human adipocytes. Similarly to major adipogenic transcription factor, PPARγ, FXRα expression was elevated with the adipogenic conversion, which does suggest an involvement of FXRα in human adipogenesis. Even early stages of adipogenesis in the presence of UDCA were accompanied with reduced expression of PPARγ, aP2 and FASN similarly to the effects of specific ligand FXR GW4064 (Figure 4C). However, the fact that GW4064 and UDCA had completely opposite effect on the expression of FXRα mRNA itself does not support the assumption that UDCA inhibitory effect on adipogenesis might be dependent on activation of FXR. 

Surprisingly, while TUDCA presence during differentiation of cells did not modify expression of PPARγ and other genes involved in lipid handling, it reduced expression of aP2 (Figure 3D). This selective effect on aP2 without impact on adipogenesis and lipid accumulation is potentially beneficial, as inhibition of aP2 activity in mice decreased macrophage infiltration and inflammation in AT [41].

Another known bile acid receptor, TGR5, is preferentially expressed in brown AT, skeletal muscle and immune cells [42] and very recently it has been found also in whole white AT [43]. In addition to these previous findings, we have detected TGR5 mRNA in both sAAT and sGAT adipocytes/preadipocytes (Figure 3A). TGR5 mRNA levels were strongly elevated when adipogenic medium containing IBMX and rosiglitazone was used. It was therefore possible that not the adipogenesis itself but one of these compounds induces TGR5 expression. However, short exposition of cells to differentiation medium lacking either dexamethasone or IBMX with rosiglitazone did not stimulate TGR5 expression (not shown). Therefore full activation of adipogenic process is necessary for upregulation of TGR5 expression. Activation of TGR5 in brown AT and skeletal muscle leads to increased UCP1 activity, oxidative phosphorylation and energy expenditure [12,44]. However, presence of BAs during the differentiation of preadipocytes did not enhance UCP1 expression, quite contrary, UDCA strongly inhibited both TGR5 and UCP1 expression (Figure 4C). Therefore, the lower accumulation of lipids in adipocytes treated with UDCA cannot be ascribed to enhanced energy expenditure through mitochondrial uncoupling.

In Figures 3D and 4C, we shown that addition of UDCA to adipogenic medium suppressed PPARγ expression. In order to provide possible mechanism of UDCA on adipogenesis, we investigated activity of Erk1/2 that is both upstream of PPARγ induction [45] and putatively activated by UDCA [16]. Erk1/2 activation has both pro- and anti-adipogenic outcomes, depending on the exact timing [45]. Brief activation or Erk1/2 is in 3T3-L1 prerequisite for mitotic clonal expansion and when it is prevented in this specific time window, 3T3-L1 fail to differentiate into adipocytes [46]. In contrast, prolonged activity of Erk1/2 is linked with lowered PPARγ transcriptional activity [47] and supports osteogenic differentiation pathway in expense of adipogenic program [48]. We brought the evidence that Erk1/2 phosphorylation is sustained in the presence of UDCA for at least 24 hours, when it was minimal in control cells. Thus, it can be suggested that UDCA may lower sensitivity of cells to adipogenic stimuli by Erk1/2 – dependent inhibition of PPARγ.

Finally, we tested whether UDCA and TUDCA may decrease expression of cytokines by adipocytes and thus inhibit attraction of monocytes and macrophages into AT. Exposition to BAs did not lead to reduction of expression of cytokines in adipocytes. On the contrary, UDCA strongly upregulated mRNA levels of IL8, GROα, MCP1 and TLR4 in sGAT adipocytes. The mechanism of UDCA-induced expression of chemokines in adipocytes remains, however, to be investigated, because the classic inflammatory pathway including NFκB activation was unaltered by UDCA treatment in human preadipocytes, similarly as was shown earlier in cancer cells [49]. Selective activation of IL8 and TLR4 by UDCA in sGAT adipocytes was rather unexpected. It could suggest that sGAT adipocytes may under certain stimulation attract more macrophages compared to sAAT adipocytes. Recently published comparison of sAAT and sGAT depots revealing higher expression of pro-inflammatory cytokines and macrophage markers in sGAT [50] supports this hypothesis. 

Together, our data showed that UDCA inhibits both proliferation and differentiation of human preadipocytes derived from two distinct subcutaneous AT depots. Therefore the potential therapeutic use of UDCA different from treatment of cholestatic diseases should be considered with caution. Nevertheless, its taurine conjugate TUDCA does not have the same negative impact on the function of human preadipocytes and it lowers partially the demands on ER function. Thus, we suggest TUDCA as a preferred chemical chaperone for modulation of ER function in vivo. 
